# Tuberculosis treatment survival of HIV positive TB patients on directly observed treatment short-course in Southern Ethiopia: A retrospective cohort study

**DOI:** 10.1186/1756-0500-5-682

**Published:** 2012-12-12

**Authors:** Debebe Shaweno, Alemayehu Worku

**Affiliations:** 1 School of Public and Environmental health, Hawassa University, P.O.Box 1560, Hawassa, Ethiopia; 2 Associate professor of Biostatistics, School of public health, College of Health Sciences Addis Ababa University, P O Box 32812, Addis Ababa, Ethiopia

**Keywords:** TB treatment, Survival, TB patients, HIV positive, Hawassa, Health center

## Abstract

**Background:**

Tuberculosis (TB) and HIV co-infection remains a major public health problem. In spite of different initiatives implemented to tackle the disease, many countries have not reached TB control targets. One of the major attributing reasons for this failure is infection with HIV. This study aims to determine the effect of HIV infection on the survival of TB patients.

**Findings:**

A retrospective cohort study was employed to compare the survival between HIV positive and HIV negative TB patients (370 each) during an eight month directly observed treatment short-course (DOTS) period. TB patient’s HIV status was considered as an exposure and follow up time until death was taken as an outcome. All patients with TB treatment outcomes other than death were censored, and death was considered as failure. Cox proportional hazard regression model was used to determine the hazard ratio (HR) of death for each main baseline predictor. TB/HIV co-infected patients were more likely to die; adjusted Hazard Rate (AHR) =1.6, 95%CI (1.01, 2.6) during the DOTS period. This risk was statistically higher among HIV patients during the continuation phase (p=0.0003), as a result HIV positive TB patients had shorter survival (Log rank test= 6.90, df= 2, p= 0.008). The adjusted survival probability was lower in HIV positive TB patients (< 15%) than HIV negative TB patients (> 85%) at the end of the DOTS period (8^th^ month).

**Conclusion:**

TB treatment survival was substantially lower in HIV infected TB patients, especially during the continuation phase. Targeted and comprehensive management of TB/HIV with a strict follow up should be considered through the entire TB treatment period.

## Findings

## Background

Tuberculosis and HIV co-infection remains a major public health challenge throughout the world. An extra 25% of deaths among TB patients is attributable to co-infection with HIV according to the WHO 2009 TB report [1]. TB is often the first opportunistic infection and a leading cause of death in HIV infected persons [2-4].

HIV infection is the primary reason for the failure to meet tuberculosis control targets (at least 85% cure rate among new sputum smear positive TB cases) [5] in countries with high HIV infection [6]. This is attributable to factors such as over diagnosis of sputum smear-negative TB, under diagnosis of sputum smear-positive TB, low cure rates, high morbidity, mortality and default rates during treatment [6,7], and atypical clinical presentation of TB in HIV infected patients [8]. Consequently, HIV-infection leads to diagnostic challenges and delays in identifying TB that profoundly impacts treatment outcome [8]. Ethiopia ranks seventh among the world’s 22 high-burden TB countries [9,10].

In Ethiopia in 2007, TB was the cause of 76,000 deaths, of which 30% were among HIV positive patients [1,9]. HIV infected people are more likely to have severe forms of TB (disseminated and extra-pulmonary), as a consequence of which TB is the leading cause of mortality and morbidity in HIV infected people, with up to one in three dying from TB [11].

Many studies and reports elsewhere have demonstrated a high TB treatment death rate in TB/HIV co-infected patients when compared to HIV negative TB patients[1,12], as high as 35%.

The goal of TB/HIV collaborative activities is to reduce mortality, default and relapse, and to prevent drug resistance [5,6,13,14]. Only few studies showed the critical time of death, but with questionable power [15]. The present study assessed the impact of HIV infection on the survival of TB patients during the period of DOTS in southern Ethiopia.

## Methods

This study was conducted in February 2010 to determine the impact of HIV infection on the survival of TB patients treated from 2006–2010 in Hawassa Health Center (HHC) which is located in Hawassa, the capital city of the Southern Nations Nationalities and Peoples Region (SNNPR) of Ethiopia.

As per the national guidelines for management of TB [5], patients with symptoms suggestive of pulmonary TB submit three (spot, morning, spot) sputum samples. If at least two smear results were positive, a patient was regarded as smear positive. Since errors made during handling of the specimen can never be excluded, only one positive smear result in HIV negative patient does not confirm diagnosis of TB. Therefore, diagnosis of smear positive pulmonary TB in patients with only one positive smear result was supported by radiologic abnormalities consistent with active pulmonary tuberculosis. In HIV positive patients, one positive smear result was necessary to make diagnosis of smear positive pulmonary TB. Similarly, a patient who had negative smear results and showed no progress following treatment with non-specific broad-spectrum antibiotics (excluding anti-TB drugs) for suspected bacterial infection was categorized as having smear negative pulmonary TB.

Extra-pulmonary tuberculosis (EPTB) was not diagnosed in the health center but in hospitals clinically by decision of the clinicians. Patients with visible external masses were requested to have biopsy tests.

Patients diagnosed with TB were referred to TB clinics to receive an HIV test and be registered for DOTS. A TB patient who comes to the TB clinic for treatment undergoes a process called provider initiated HIV counseling and testing, where the TB care provider counsels the patient for HIV test. A rapid test algorithm is used to determine HIV infection***.*** The patient’s HIV status and other relevant information including treatment outcome were recorded on the TB log book prescribed by the Ministry of Health of Ethiopia.

Smear positive patients registered in the DOTS clinic receive 8 months short course chemotherapy: daily supervised ethambutol, rifampicin, isoniazid and pyrazinamide (ERHZ) combination for 2 months followed by self-administered ethambutol and isoniazid (EH). Smear negative and EPTB patients receive rifampicin, isoniazid and pyrazinamide (RHZ) during the intensive phase and EH during the continuation phase. According to the national TB treatment guideline, new smear positive cases, seriously ill smear negative cases and seriously ill new EPTB cases were placed under TB treatment category one; whereas new smear negative and new EPTB cases who were not seriously ill were treated under TB treatment category three [5].

Before data collection, two independent sampling frames were developed by taking HIV status of TB patients as a stratifying variable. The sampling frame for HIV positive stratum constituted 420 TB patients and that for HIV negative stratum constituted 1,350 TB patients registered during the study period. This retrospective cohort study compared TB treatment survival during an eight month DOT period between 370 HIV positive and 370 HIV negative TB patients randomly selected from each sampling frame using a computer generated random numbers.

Survival time was months from the date of initial diagnosis of TB to death while in TB treatment or, in the case of individuals who did not die, the last follow-up recorded by the health center. Whereas individuals who died were considered as failures, those who remained alive until the end of the treatment or dropped out of treatment were considered as censored.

The sample size was determined by setting type one error at 5%, power at 80% and the exposed to non-exposed ratio at 1.0. The findings of related studies were also considered in determining sample size [1,12,16]. Data were abstracted using checklists developed by the investigators. To minimize data entry errors EPI Info version 3.3.2 was used. Data were analyzed using Stata version 9.2.

P values < 0.05 and confidence level of 95% were considered to indicate statistical significance. Cox proportional hazard regression model was used to determine the hazard ratio (HR) of death for each main baseline predictor. To assess the association between baseline variables and mortality, two strategies were used. First, each baseline variable that did not violate assumptions was entered into a separate Cox proportional hazards model. Second, a multivariate Cox proportional hazards model was fitted with the predictors that have P ≤ 0.3 in the bivariate model. Eventually only those variables that remained significant at p ≤ 0.05 in the final model were retained as independent predictors of survival. Life table survival analysis was used to estimate DOT survival probability of TB patients. Kaplan Meier analysis was also used to estimate graphically the survival probabilities. The observed difference in survival time between HIV positive and HIV negative TB patients was compared using the log rank test. Violation of proportional hazard assumptions was checked by procedures: Log (−log (st) plots, Schoenfeld residual plots and by regressing Schoenfeld residuals against time to test for independence between time and residuals. Incidence rates with person-months and cumulative incidence were calculated for death as TB treatment outcome.

The study protocol was approved by the Institutional Review Board (IRB) of Addis Ababa University, College of Health Sciences.

## Results

### Description of the cohort

Among TB patients registered for the DOT program from 2006 –2010, the data of 740 (370 HIV positive and 370 HIV negative) patients were retrieved from the TB logbook for the study. Nearly all of the study subjects; 99.2% of HIV positive and 96.2% of HIV negative TB patients were from urban areas. The median and inter quartile range(IQR) for the age of HIV positive TB patients were 30 and 25–36 years respectively while the corresponding values for HIV negative study subjects were 25 and 20–36 years. Males were predominant in both groups especially among HIV negative TB patients; 189 (51.1%) in HIV positives and 228 (61.6%) in HIV negatives. Equal proportions of TB patients were treated for TB as new cases in both HIV positive 351 (94.9%) and negative 345 (93.2%) study cohorts.

A higher proportion of HIV positive 351 (94.9%) than negative 322 (87%) TB patients were treated as WHO TB treatment category one. But a lower proportion of HIV positive 4 (1.1%) than negative 25 (6.8%) TB patients were treated as WHO TB treatment category three.

Among HIV positive TB patients, 124 (33.24%) were known to have started cotrimoxazole prophylactic therapy (CPT). Regarding anti retroviral treatment (ART) initiation, out of 370 HIV positive TB patients; 52 (14.1%) were known to have started, 39 (10.5%) were known not to have started, and the rest 279 (75.4%) had no known history of ART initiation. Information on marital status was recorded for only 130 (35.1%) HIV positive patients, of whom 56 (43.1%) were single. Similarly, of 225 (60.8%) HIV negative TB patients with recorded marital status, 138 (61.3%) were single. Regarding TB type, 21.6% of HIV positive and 47% of HIV negative TB patients had smear positive pulmonary TB (Table 1).

**Table 1:** Baseline characteristics of TB patients treated in Hawassa health center from 2006–10

**Baseline variables**	**HIV positive(n=370)**	**HIV negative(n=370)**	**Total**
Residence			
Urban	367(99.2)	356(96.2)	723(97.7)
Rural	3(0.8)	14(3.8)	17(2.3)
Sex			
Male	189(51.1)	228(61.6)	417(56.4)
Female	181(48.9)	142(38.4)	323(43.6)
Age (years)			
15- 20	39(10.5)	101(27.3)	140(18.9)
21-40	279(75.4)	197(53.2)	476(64.3)
41-60	48(13)	58(15.7)	106(14.3)
60-90	4(1.1)	14(3.8)	18(2.4)
Marital Status (n=355)			
Single	56(43.1)	138(61.3)	194(54.6)
Married	64(49.2)	84(37.3)	148(41.7)
Divorced	9(6.9)	2(0.9)	119(33.5)
Widowed	1(0.8)	1(0.5)	2(0.6)
no mention	240(64.9)	145(39.2)	385(52)
Case definition			
New	351(94.9)	345(93.2)	696(94)
Relapse	17(5)	22(6)	39(5.3)
Return after default	2 (0.5)	10.3)	3(0.4)
Failure	0	2(0.5)	2(0.3)
TB Type			
Pulmonary positive	80(21.6)	177(47.8)	257(34.7)
Pulmonary negative	234(63.2)	115(31.1)	349(47.2)
EPTB	56(15.1)	78(21.1)	134(18.1)
Rx Category			
I	351(94.9)	322(87)	673(91)
II	15(4.1)	23(6.2)	38(5.1)
III	4(1.1)	25(6.8)	29(3.9)
Diagnosis Center			
Hawassa Health center	275(74.3)	263(71.1)	538(72.7)
Hospital	88(23.8)	103(27.8)	191(25.8)
Others	7(1.9)	4(1.1)	11(1.5)
CPT initiated (n=133)			
Yes	123(92.5)		123(92.5)
No	10(7.5)		10(7.5)
No mention	237(64.1)		237(64.1)
ART Initiated (n=91)			
Yes	52(14.1)		52(14.1)
No	39(10.5)		39(10.5)
No mention	279(75.4)		279(75.4)

CPT= Cotrimoxazole, ART=Antiretroviral Therapy.

### Time of occurrence for death

A total of 78 deaths occurred during the TB treatment period. Most of the deaths 46 (58.9%) [24 in HIV positive and 22 in HIV negative] occurred during the intensive phase of treatment. The majority of deaths 50 (64%) occurred in HIV positive TB patients. In accord with the above finding, the mean and median time of death for those who died in the intensive phase was 27.9 and 29 days respectively.

Concerning the association between HIV status and risk of death during the intensive phase, the risk does not significantly differ between HIV positive and HIV negative TB patients (p=0.15). Unlike the situation during the intensive phase, the risk was higher among HIV positive TB patients during the continuation phase, 7.5 per 1000 (26 of 346) compared to HIV negative TB patients 1.7 per 1000 (6 of 348) (p=0.0003). The incidence rate of death among HIV positive study subjects was 20.6 per 1000 person months of follow up (50 died in a 2430.2 months of follow up) whereas the rate was 10.8 per 1000 person months of follow up (28 died in a 2582.6 months of follow up) among HIV negative TB patients. If one is HIV positive and on TB treatment the risk of death was 2 times higher than for an HIV negative patient on TB treatment: RR (95%CI) 1.9[1.2- 3.1]. Almost half of the deaths among HIV positive TB patients were attributable to HIV infection: AR% (95% CI) 47.3[14.7-68.1]. The PAR% was 30.3%.

### Survival status of the study subjects

Of 370 HIV positive and 370 HIV negative TB patients followed for 8 months, 50 (13.5%) of the HIV positive and 28 (7.6%) of the HIV negative died and were treated as failure in the analysis. The remaining 320 (86.5%) HIV positive and 342 (92.4%) HIV negative patients became censored during the follow up period. The minimum follow up period was 1 and 3 days in HIV positive and HIV negative TB patients respectively, with the maximum being 8 months for both groups. The majority of deaths, 46 (58.97%) occurred in the intensive phase (the first 2 months) of treatment, whereas the rest of the deaths, 32 (41.03%), occurred during the continuation phase (6 months).

Life table analysis was used to estimate the survival probabilities of TB patients. The rate of survival at the end of the intensive phase was the same in HIV positive and HIV negative TB patients (93.7% and 94.0%, respectively). After the intensive phase, the survival rate in HIV positive TB patients declined sharply to 85.7% within seven months of treatment, and then remained at 85.7% until all patients become censored at month eight. But in HIV negative TB patients, at the end of the 8^th^ month, by which time all patients were censored, the survival rate was 92.2%, (Table 2).

**Table 2:** Survival of TB patients by HIV status, Hawassa health center 2006–2010

	**HIV Positive**				**HIV Negative**			
**Interval (month)**	**N**	**Death**	**Censored**	**Survival**	**N**	**Death**	**Censored**	**Survival**
(0–1)	370	10	7	0.9727	370	13	5	0.9646
(1–2)	353	13	7	0.9365	352	9	8	0.9397
(2–3)	333	10	6	0.9082	335	1	4	0.9369
(3–4)	317	5	8	0.8936	330	1	7	0.934
(4–5)	304	8	14	0.8696	322	2	7	0.9281
(5–6)	282	2	9	0.8633	313	2	6	0.9221
(6–7)	271	2	3	0.8569	305	0	7	0.9221
(7–8)	266	0	1	0.8569	298	0	2	0.9221
(8–9)	265	0	265	0.8569	296	0	296	0.9221

**Figure 1:**
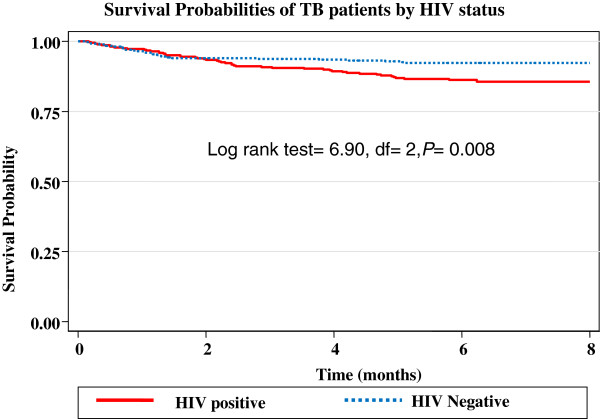
Survival probabilities by HIV status of TB patients treated in Hawassa health center, 2006-2010.

In agreement with the life table estimation, the Kaplan Meier survival curve (Figure 1) showed statistically lower survival in HIV positive TB patients (log rank statistic= 6.90, df= 2, P= 0.0086).

**Figure 2:**
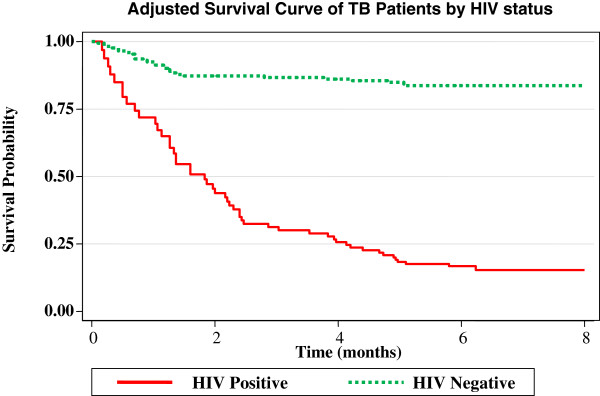
Adjusted survival curve of the study subjects by HIV status, Hawassa Health Center, 2010.

In the adjusted survival curve (adjusted to baseline characteristics in table one), the survival rate of HIV negative TB patients was > 85% at the end of eighth month. But for HIV positive TB patients, the median survival was at about the end of intensive phase, and dropped to < 15% at the end of the study period (8^th^ month) where all the remaining TB patients become censored (Figure 2).

### Factors affecting the survival of TB patients on DOTs

The relationship between the main baseline variables and the risk of death was analyzed using a Cox proportional hazard regression model. Before fitting the covariates into the model, proportional hazard assumption were checked by plotting “Schoenfeld residuals, regressing Schoenfeld residuals against time to test for independence between time and residuals and by examining log (log (st) plots. The covariates which violated the assumptions were not included in the Cox model. The result showed that age, weight, smear negative pulmonary TB, dose of anti TB drugs, and HIV status were all significantly associated with death of TB patients during the period of DOT (Table 3).

**Table 3:** Univariate predictors of death among TB patients on DOTs in Hawassa health center, 2006–10

**Covariates**	**Number at risk**	**Number of death**	**Crude hazard ratio**	**95% CI**	**P-value**
Urban	723	75	1		
Rural	17	3	1.9	(0.59,5.9)	0.28
Gender					
Male	417	43	1		
Female	323	35	1.03	(0.66-1.6)	0.14
Age(years)	740	78	1.028	(1.01,1.04)	0.000
Weight(kg)	740	78	.944	(0.92, .97)	0.000
Marital Status(n=342)					
Single	194	16	1		
Married	148	22	1.9	(0.99,3.6)	0.053
Center TB Diagnosed					
Health Center	549	59	1		
Hospital	191	19	0.92	(0.55,1.54)	0.74
TB treatment history					
New	696	73	1		
Re-treatment	44	5	1.1	(0.43,2.6)	0.9
Treatment Category					
I	673	72	1		
II	38	4	0.97	(0.35,2.6)	0.9
III	29	2	0.61	(0.15,2.5)	0.5
Smear(baseline)n=606					
Positive	257	20	1		
Negative	349	47	1.8	(1.1,3.1)	0.022
HIV Status					
Negative	370	28	1		
Positive	370	50	1.84	(1.2, 2.9)	0.01
CPT(n=133)					
No	10	2	1		
yes	123	14	0.38	(0.09,1.7)	0.21
ART(n=91)					
No	39	7	1		
yes	52	9	0.88	(0.33,2.4)	0.8

Compared to HIV negative patients, the HR of dying from TB increased significantly by 60% in HIV positive TB patients on DOTS; AHR = 1.6, 95% CI: [1.01, 2.6]. Mortality risk among TB patients increases by about 3% for every year increase in age; AHR =1.03, 95% CI: [1.02,1.05]. Accordingly, for every five and 10 years increase in age, the risk of death increases by 15.9% and 34.4% respectively. However, mortality risk decreases by about 6% for every kilogram (kg) increase in weight. This risk further decreases by 26.6% for every five kg increase in weight (Table 4).

**Table 4:** Multivariate cox regression model of factors associated with death of TB patients treated in Hawassa health center from 2006–2010

**Covariates**	**Number at risk**	**Number of deaths**	**Adjusted hazard ratio**	**95% CI**	**P-value**
Residence					
Urban	723	75	1		
Rural	17	3	1.96	(0 .6, 6.4)	0.26
Gender					
Male	417	43	1		
Female	323	35	0.87	(0.51, 1.5)	0.61
Age(years)	740	78	1.03	(1.02,1.05)	0.000
Weight(kg)	740	78	0.94	(0.9, 0 .97)	0.000
TB Type					
Smear positive PTB	257	20	1		
Smear positive PTB	349	47	1.2	(0.68,2.1)	0.53
EPTB	134	11	0.9	(0.4, 1.9)	0.84
HIV Status					
Negative	370	28	1		
Positive	370	50	1.6	(1.01, 2.6)	0.04

A multivariate Cox proportional hazard adjusted model was fitted with variables having a p-value < 0.3 in the bivariate Cox proportional model. However, cases with missing values were excluded from the multivariate analysis. In addition, the smear result at baseline was dropped because of its colinearity with other predictors. In the final model, variables with a p-value less than 0.05 were retained as independent predictors of death. Accordingly, only three covariates, namely being HIV positive, age, and baseline weight remained as independent predictors of death (Table 4).

## Discussion

Death caused by TB is preventable. Management can be modified at the time of highest risk for death if known. Many researchers outside Ethiopia have discussed higher TB mortality in HIV positive TB patients. Ethiopia does have no such data [1], yet the TB/HIV co- infection is among the highest in Africa. This study has discussed the effect of HIV infection on the survival of TB patients during DOTS period in Ethiopia by comparing survival difference between HIV positive and negative TB patients using a power of 87%. Similarly, the study has demonstrated significant difference on the time of death due to HIV infection.

Similar to the current study, numbers of studies [14,17] have reported higher overall death rates among HIV-positive TB patients; as high as four times increased risk of death in a previous report during TB treatment. In this study, almost half of the deaths in HIV positive TB patients were attributed to their HIV infection. If HIV positive TB patients had not been infected by HIV, almost 50% of the observed deaths would not have occurred.

Information about the time of death among TB patients while on treatment could help provide the necessary care timely. In the current study, irrespective of one’s HIV status, most of the deaths occurred during the intensive phase. This may suggest that late presentation was a major factor for early death than HIV infection itself.

Unlike another study elsewhere [15], risk of death was not different in the intensive phase between HIV positive and negative TB patients, but significantly higher among the HIV positive TB patients during the continuation phase. This finding may suggest the self supervised treatment for a longer period (6 months) in the TB/HIV co-infected has some limitation (adherence might have been a problem in HIV/TB co- infected).

In the current study, the survival probability is significantly lower in HIV positive TB patients; internally consistent with the earlier finding that significantly more deaths occurred in HIV positive TB patients. In addition, when factors such as age, sex, TB type, baseline weight and age were kept constant, the median survival probability of HIV positive TB patients would be within intensive phase and eventually reaches to less than 15% at the end of continuation phase, while the survival for HIV negative TB patients remains above 85% throughout the course of DOTS.

In keeping with previous studies [17], age, baseline weight and HIV infection were found to be independent predictors of [7] death among TB patients on DOTS in a multivariate adjusted Cox model. Studies showed that risk of death from TB increases in individuals with weight loss. Weight loss is a very common phenomena in HIV (to the extent of being considered as AIDs defining illness) and TB patients [18].

Weight loss is much pronounced in patients with dual infection (TB/HIV co infected) compared to HIV negative TB patients [19]. Consistent with the studies mentioned, and internally with Cox model, baseline weight was significantly lower in HIV positive TB patients. AIDs patients were likely to become malnourished from constantly being sick, from diarrhea that prevents absorption of nutrients, from loss of appetite and sores of mouth that make eating difficult and from opportunistic infections. Similarly, weight loss in TB patients might be explained partly by the loss of appetite and loss of energy by the disease itself [20].

Various studies outside Ethiopia have indicated that the risk of dying from TB increases with the patient's age. Evidences showed that TB is among the five top killers of geriatric population. Mortality from TB in older individuals is attributed to the increased physiologic risk of death, to the vague symptoms in the elderly, diagnostic problems (the problem may be overlooked in elderly because of other conditions that preoccupy the attention of physicians) and presence of co-morbidities and difficulties in accessing diagnosis in this group of patients, thereby delaying the onset of treatment [21,22].

In congruence with the above studies, in the present study, the relative risk of dying from TB increased with the age of individuals in a dose–response manner in both the univariate and multivariate analysis.

Apart from such important findings, this study is not without limitation. As common for secondary data studies, important variables; CD4 level, ART initiation and CPT initiation were almost incomplete from the records.

## Conclusions

The study demonstrated substantially lower survival probability for HIV positive TB patients during TB treatment period. The impact of HIV infection was magnified during continuation phase; higher death was demonstrated in HIV positive TB patients during the self administered treatment period (continuation phase) compared to HIV negative TB patients. TB treatment survival of TB patients is lowered when one is HIV positive, is in an advanced age category and has low baseline weight.

### Recommendations

HIV positive TB patients need strict follow up not only during the intensive phase but also during the continuation phase. Alongside with the treatment of both infections, mandatory nutrition counseling should be part and parcel of TB management in patients with weight loss subsequent to investigating the possible causes of weight loss. The management of TB in HIV positive and elderly patients should be differentiated and comprehensive enough.

## Competing interests

The authors declare that they have no competing interests.

## Authors’ contribution

DS conceived the study, formulated the design, drafted the manuscript, analyzed and interpreted the data. AW participated in the conception of the study and revised the manuscript critically for important intellectual content. All authors have read and approved the final manuscript.
